# Supplementary material for: The Systems Biology Research Tool: evolvable open-source software
Source: BMC Syst Biol. 2008 Jun 29;2:55. doi: 10.1186/1752-0509-2-55 (PMC2446383; doi:10.1186/1752-0509-2-55)
Supplement: Additional file 1 — SBRT Archive. An archive of the current version of the Systems Biology Research Tool. [file 1752-0509-2-55-S1.zip › sbrt-1.4.0/doc/developers_guide/api/sbrt/kernel/math/geom/comp/package-summary.html]

sbrt.kernel.math.geom.comp


|  |  |  |
| --- | --- | --- |
| **Overview** \| **Package** \| Class \| **Use** \| **Tree** \| **Deprecated** \| **Index** \| **Help** | |  |
| **PREV PACKAGE**   **NEXT PACKAGE** | **FRAMES**    **NO FRAMES**     **All Classes** |  |


---

## Package sbrt.kernel.math.geom.comp

Provides classes and interfaces for performing geometry-related computations.

**See:**
  
          **Description**

| **Class Summary** | |
| --- | --- |
| **CdHarComputer<V>** | This class is an implemenation of a coordinate-direction hit-and-run algorithm. |
| **EuclideanDist** | This class is used to compute Euclidean distances. |
| **Hamming** | This class is used to compute Hamming distances - the number of differences between two "strings" of equal length. |
| **Taxicab** | This class is used to compute Taxicab distances. |

## Package sbrt.kernel.math.geom.comp Description

Provides classes and interfaces for performing geometry-related computations.

---


|  |  |  |
| --- | --- | --- |
| **Overview** \| **Package** \| Class \| **Use** \| **Tree** \| **Deprecated** \| **Index** \| **Help** | |  |
| **PREV PACKAGE**   **NEXT PACKAGE** | **FRAMES**    **NO FRAMES**     **All Classes** |  |


---
